# Burden, causes, and outcomes of people with epilepsy admitted to a rural hospital in Kenya

**DOI:** 10.1111/epi.12935

**Published:** 2015-02-16

**Authors:** Symon M. Kariuki, Eddie Chengo, Fredrick Ibinda, Rachael Odhiambo, Anthony Etyang, Anthony K. Ngugi, Charles R. J. C. Newton

**Affiliations:** ^1^ KEMRI‐Wellcome Trust Research ProgrammeKilifiKenya; ^2^ Nuffield Department of MedicineUniversity of OxfordOxfordUnited Kingdom; ^3^ Research Support UnitFaculty of Health SciencesAga Khan University of East AfricaNairobiKenya; ^4^ Department of PsychiatryUniversity of OxfordOxfordUnited Kingdom

**Keywords:** Incidence, Disability‐adjusted life years, Causes, Outcome, People with epilepsy, Hospital admissions, Kenya

## Abstract

**Objective:**

People with epilepsy (PWE) develop complications and comorbidities often requiring admission to hospital, which adds to the burden on the health system, particularly in low‐income countries. We determined the incidence, disability‐adjusted life years (DALYs), risk factors, and causes of admissions in PWE. We also examined the predictors of prolonged hospital stay and death using data from linked clinical and demographic surveillance system.

**Methods:**

We studied children and adults admitted to a Kenyan rural hospital, between January 2003 and December 2011, with a diagnosis of epilepsy. Poisson regression was used to compute incidence and rate ratios, logistic regression to determine associated factors, and the DALY package of the R‐statistical software to calculate years lived with disability (YLD) and years of life lost (YLL).

**Results:**

The overall incidence of admissions was 45.6/100,000 person‐years of observation (PYO) (95% confidence interval [95% CI] 43.0–48.7) and decreased with age (p < 0.001). The overall DALYs were 3.1/1,000 (95% CI, 1.8–4.7) PYO and comprised 55% of YLD. Factors associated with hospitalization were use of antiepileptic drugs (AEDs) (odds ratio [OR] 5.36, 95% CI 2.64–10.90), previous admission (OR 11.65, 95% CI 2.65–51.17), acute encephalopathy (OR 2.12, 95% CI 1.07–4.22), and adverse perinatal events (OR 2.87, 95% CI 1.06–7.74). Important causes of admission were epilepsy‐related complications: convulsive status epilepticus (CSE) (38%), and postictal coma (12%). Age was independently associated with prolonged hospital stay (OR 1.02, 95% CI 1.00–1.04) and mortality (OR, 1.07, 95% CI 1.04–1.10).

**Significance:**

Epilepsy is associated with significant number of admissions to hospital, considerable duration of admission, and mortality. Improved supply of AEDs in the community, early initiation of treatment, and adherence would reduce hospitalization of PWE and thus the burden of epilepsy on the health system.

Epilepsy is a common neurologic disorder directly affecting about 70 million people in the world, with about 90% residing in lower‐and middle‐income countries.^1^ The treatment gap for epilepsy is large (up to 90%) in these areas, and this is in part due to negative attitudes toward biomedical services, cost of accessing diagnosis and treatment, and traditional beliefs about the cause of epilepsy.^2^, ^3^ People with epilepsy (PWE) are thought to have more hospital admissions than those with other chronic neurologic disorders,^4^ and is the third most common after stroke and spinal cord injuries in sub‐Saharan Africa (SSA).^5^

Epilepsy‐related hospital admissions are increasing in high‐income countries; for example, the rate of hospital use increased from 66 to 68/100,000 persons/year over 10 years in England and Wales,^4^ and by 27% over 12 years in the United States.^6^ In the American study, epilepsy accounted for more than half of all hospital stays among admissions of PWE, with respiratory‐related complications being the most common causes for admission.^6^ The hospital admission rates would be expected to be higher in SSA compared to the West because of the higher prevalence of epilepsy^7^ and the larger treatment gap.^3^ In addition, epilepsy in Africa is associated with increased complications such as status epilepticus, burns, and head injuries,^8^ which often require treatment at the hospital. Nevertheless, there are few published studies that document the burden, causes, and duration of hospitalization in PWE in SSA, where epilepsy may add a considerable burden on the health system. In Zambia PWE had a poorer socioeconomic status compared to their peers with other medical conditions,^9^ but the focus of this study was not to examine the burden of hospitalization due to epilepsy.

This study estimates the incidence of admissions with epilepsy, the associated disability‐adjusted life years (DALYs) and determines the important causes and risk factors of admission to a rural hospital among PWE. We further examined the risk factors associated with the major outcomes (duration of hospitalization and mortality) among PWE.

## Methods

### Study site and population

The study was conducted at Kilifi County Hospital (KCH), the only district level hospital in a rural area with about 250,000 residents. The hospital has a bed capacity of 172 patients and provides general medical and surgical services. An epilepsy clinic has been running since 2003, but many patients visit the psychiatric clinic at KCH, peripheral health centers, and private clinics.

Most of the people in the district are poor and earn a living either through peasant farming (about 70%) or fishing.^10^ The prevalence of epilepsy in the community is high.^7^ The treatment gap remains a challenge, although it has dropped from 70% in 2003^11^ to 62% in 2008 following health education and sensitization programs in the community,^12^ and to 33% in those prescribed antiepileptic drugs (AEDs) following the introduction of the Kilifi Epilepsy Education Programme (KEEP).^13^ Most patients are treated with standard AED such as phenobarbital, phenytoin, carbamazepine, and sodium valproate, according to the national guidelines.^14^ Cultural beliefs influence the use of biomedicine in the management of epilepsy.^15^

### Selection of study participants and data extraction

This study includes all children and adults admitted with epilepsy‐related complications to KCH. Children (those 13 years or younger), are admitted to the pediatric ward and those older are admitted to the adult wards. We reviewed the admissions register from January 2003 through to December 2011 for patients with a diagnosis of epilepsy and/or history of AED use. The available medical notes were reviewed manually and the following information was obtained: age, sex, address, duration of hospital stay, secondary and tertiary diagnoses and causes of admission (nature of injuries and other ailments), outcomes, and use of AEDs and other drugs. Patients excluded from the study were those for whom there were insufficient data regarding the diagnosis or incorrect diagnosis of epilepsy. The data were extracted in a standard proforma and prepared for statistical analysis by SK and EC, with any disagreements (about 5%) resolved through consensus and in consultation with CN (a pediatric neurologist).

### Case definitions

Epilepsy was defined as a history of at least two unprovoked seizures according to the International League Against Epilepsy (ILAE) criteria,^16^ excluding all acute symptomatic seizures meeting a criteria defined previously.^17^ We categorized the causes of admission as follows; convulsive status epilepticus (CSE); burns; accidents (epilepsy related falls and fractures); postictal coma (history of seizure immediately prior to admission; with a summated Blantyre Coma Scale ≤2 in children older than 9 months^18^ and Glasgow Coma Scale score ≤8 in adults); febrile illnesses such as meningitis, malaria, and respiratory infections; postictal psychosis (defined as visual or auditory hallucinations post seizures); anemia; poisoning; hypertension; skin infections; and diarrheal disease, among others.

CSE was defined as continuous seizures lasting ≥30 min and associated with impaired consciousness or repetitive seizures lasting ≥30 min with impaired consciousness in between the seizures.^19^ Also classified as probable status epilepticus were those patients who required parenteral anticonvulsants (phenobarbital and/or phenytoin) to stop continuing seizures (i.e., seizures lasting more than 5 min).^20^ Seizure semiology was classified as generalized or focal (including focal becoming generalized).^21^ Nonadherence to AEDs was based on self‐reports. The main outcomes were mortality and prolonged hospital stay, which was defined as a duration of admission ≥75th percentile, representing the extreme or top quartile distribution.

### Statistical analysis

Data were entered in Excel (Microsoft, Redmond WA, U.S.A.) and transferred to STATA (Version 11; Stata Corp, Lakeway Drive College Station, TX, U.S.A.) for analysis. Categorical variables were compared with Pearson's chi‐square test or Fisher's exact test, where the expected cell frequency was less than five counts. Continuous variables were compared using student *t*‐test or Mann–Whitney *U*‐test, where data were nonparametric.

The factors associated with hospital admissions were determined by comparing the medical histories of those hospitalized with epilepsy with those with epilepsy who were not hospitalized; both groups were identified from a community survey conducted in 2008 in the same area using a standard methodology and questionnaire.^7^ Based on the 2008 survey, the people with epilepsy hospitalized (N = 87) and the comparison group of those not hospitalized (N = 679) were similar by age (p = 0.098), sex (p = 0.379), marital status (0.445), education (p = 0.733), and employment status (p = 0.698). The factors associated with prolonged hospital stay and mortality were determined by comparing the clinical features of those with the outcome measure of interest with those without. Associations were determined using logistic regression, in which variables with a p < 0.25 in the univariate analysis were used to build a multivariable model, adjusted for age, sex and marital status, education, and employment status, in a stepwise approach. A p ≤ 0·05 was considered statistically significant.

### Incidence analysis

The incidence of hospital use was calculated by dividing the observed index admissions by population years computed over exact periods of residence in the KHDSS, repeating this for specific age groups often used in epilepsy studies.^7^ We examined the incidence trend by calculating rate ratios by year or age group using Poisson regression.

### Analysis of DALYs

The DALY package in R statistical software http://users.ugent.be/~bdvleess/DALYcalculator/download-install/ was used to estimate DALYs, using incidence, mortality rate, average age at death, duration of epilepsy, and age at onset of epilepsy, consistent with the Global Burden of Diseases (GBD) criteria.^22^ Duration and age at onset of epilepsy were obtained from community studies in the same study area.^8^ The analysis was approved by the ethical review committee of the Kenya Medical Research Institute.

## Results

### General description

There were 992 admissions in PWE over the 8‐year period, of whom 743 (75%) were children (Table 1). The median age in years (interquartile range [IQR]) for all participants was 5.6 (2.8–12.2), and 4.0 (2.0–7.0) for children and 22.0 (18.0–29.0) for adults. The number of males in the study was 579/992 (58%) and there were significantly more male admissions among the children (451, 78%) than adults (128, 22%); (p = 0.010).

**Table 1:** Characteristics, causes, and outcomes of admissions with epilepsy

Characteristics	Children with epilepsy(N = 743)	Adults with epilepsy (N = 249)	Overall epilepsy (N = 992)
Age in years: median (IQR)	4.0 (2.0–7.0)	22.0 (18.0–29.0)	5.6 (2.8–12.2)
Male sex (%)	451 (60.7)	128 (51.4)	579 (58.4)
Focal seizures (%)	97 (13.1)	70 (28.1)	167 (16.8)
AED reported use (%)	51 (6.9)	178 (71.5)	229 (23.1)
Causes of admission			
Epilepsy related			
Convulsive status epilepticus (%)	263 (35.4)	113 (45.4)	376 (37.9)
Postictal coma (%)	19 (7.6)	113 (45.4)	122 (12.3)
Burns (%)	22 (3.0)	33 (13.3)	55 (5.5)
Postictal psychosis (%)	6 (0.8)	4 (1.6)	10 (1.0)
Accidents (%)	3 (0.4)	9 (3.6)	12 (1.2)
AED side effects	1 (0.1)	0	1 (0.1)
Not related to epilepsy			
Any febrile illnesses (>38.5°C) (%)	314 (42.3)	39 (15.4)	353 (35.6)
Malaria (%)	43 (5.8)	15 (7.7)	58 (6.2)
Respiratory infections (%)	94 (12.7)	1 (0.4)	95 (9.6)
Vomiting	2 (0.3)	0	2 (0.2)
Poisoning	1 (0.1)	2 (0.8)	3 (0.3)
Primary hypertension	0	1 (0.4)	1 (0.1)
Bullous erythema	0	1 (0.4)	1 (0.1)
Hydrocephalus	3 (0.4)	0	3 (0·3)
Other causes (%)^a^	0	33 (13.3)	33 (3.3)
Outcome			
Median hospital stay in days (IQR)	2.0 (1.0–5.0)	4.0 (2.0–9.0)	3.0 (1.0–6.0)
Prolonged hospital stay (%)	121 (16.3)	67 (26.3)	188 (19.0)
Case fatality (%)	7 (0.9)	20 (8.0)	27 (2.7)

AED, antiepileptic drug; IQR, interquartile range.

a Other causes included undetermined causes during admission. The causes of admissions are not mutually exclusive, and were determined by the authors by review of clinical notes independent of diagnosis at discharge. The epilepsy‐related complications as causes of admissions were present in 541/992 (55%).

### Rate of hospital admissions

**Figure 1:**
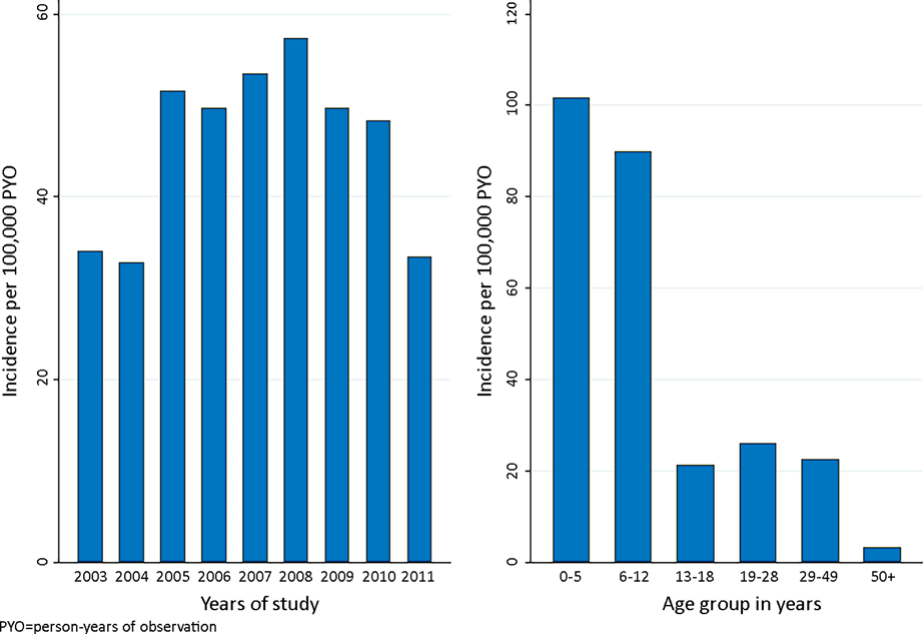
The incidence of admissions with epilepsy by years of study and age group. The incidence remained stable over the study period but decreased significantly with age group (p < 0.001).

The overall incidence of hospital admissions among PWE was 45.7/100,000 person‐years of observation (PYO) (95% confidence intervals [95% CI] 43.0–48.7), and was 32.0/100,000 PYO (95% CI 29.0–35.2) for females and 50.5/100,000 PYO (95% CI 46.6–54.8) for males, with that of males being significantly greater than for females (incidence rate ratio [IRR] 1.58, 95% CI 1.39–1.79; p < 0.001. The incidence of hospital admissions did not significantly change over the 8 years (p = 0.269), although it appeared to reduce after 2008 (Fig. 1). The incidence decreased significantly with age group (IRR 0.64, 95% CI 0.62–0.67; p < 0.001), being highest in 0–5 years (101.6/100,000 PYO, 95% CI 93.0–111.0) and lowest in 50+ years (3.2/100,000 PYO, 95% CI 1.9–5.3) (Fig. 1).

### Burden of hospital admissions among PWE

**Figure 2:**
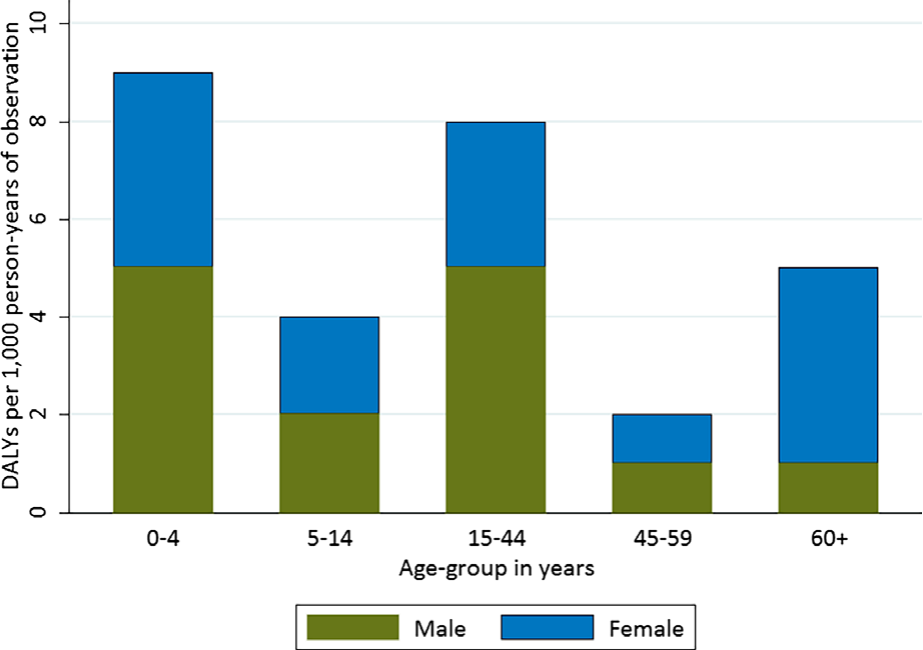
The disability‐adjusted life years displayed for five age groups and for male and female patients. The DALYs differed across age groups and between sexes.

The relative years of life lost (YLL), years lived with disability (YLD), and the DALYs for hospital admissions per 1,000 PYO were 1.6 (95% CI, 1.2–2.1), 1.5 (95% CI, 0.3–2.9), and 3.1 (95% CI, 1.8–4.7), respectively. The absolute YLL, YLD, and DALYs were 368 (95% CI, 71–720), 401 (95% CI, 287–536), and 754 (95% CI, 252–1,148), respectively. The distribution of relative DALYs by age and sex are shown in Figure 2.

### Factors associated with hospitalization

A number of medical and family history variables were associated with the hospitalization among PWE in the univariate analysis (Table 2). In the multivariable analysis, the hospitalization of epilepsy was independently associated with a history of AED use (>90% being on phenobarbital) (odds ratio [OR] = 5.26, 95% CI 2.64–10.90,; p < 0.001), previous hospitalization (OR = 11.65, 95% CI 2.65–51.17; p = 0.001), acute encephalopathy (OR = 2.12, 95% CI 1.07–4.22; p = 0.031), and adverse perinatal events (OR = 2.87, 95% CI 1.06–7.74; p = 0.038).

**Table 2:** Factors associated with admission in patients with epilepsy

Medical/family history	Univariate analysis OR (95% CI); p‐value	Multivariable analysis OR (95% CI); p‐value
Adverse perinatal events^a^	2.61 (1.15–5.93); 0.022	2.87 (1.06–7.74); 0.038
Abnormal pregnancy^a^	0.89 (0.44–1.80); 0.741	–
History of febrile seizures	1.05 (0.55–2.00); 0.889	–
Family history of any seizure	1.09 (0.65–1.82); 0.746	–
History of acute encephalopathy	2.46 (1.53–3.95); <0.001	2.12 (1.07–4.22); 0.031
Motor impairment	1.31 (0.77–2.25); 0.320	–
Previous hospitalization	5.37 (2.72–10.50); <0.001	11.65 (2.65–51.17); 0.001
Malnutrition	0.99 (0.56–1.76); 0.970	–
Learning difficulties	1.86 (1.17–2.96); 0.009	0.74 (0.32–1.70); 0.475
Neurologic deficits	1.36 (0.79–2.34); 0.275	–
Visits traditional healers	0.97 (0.59–1.59); 0.898	–
AED use	4.85 (2.91–8.06); <0.001	5.36 (2.64–10.90); <0.001
Hypertension	2.69 (0.53–13.55); 0.230	–
Burns	1.48 (0.88–2.46); 0.137	1.59 (0.68–3.73); 0.289
Head injury	0.50 (0.21–1.18); 0.114	0.45 (0.11–1.80); 0.260
Eats pork	1.48 (0.89–2.45); 0.113	1.89 (0.81–4.45); 0.142

AED, antiepileptic drugs; OR, odds ratio; CI, confidence interval.

Multivariable model included age, sex, and parental education, marital status, and employment status as covariates.

a Analysis included those with 18 years and below in whom histories for these variables are deemed reliable.

### Seizure types and treatment

Overall focal seizures were documented in 167/992 (17%) and the remainder was generalized seizures (Table 1). Focal seizures were significantly more common in adults (70/249 [28%]) than in children (97/743 [13%]); p < 0.001 (Table 1). Among children, complex seizures (focal, prolonged, and/or repetitive) were reported by 427/743 (58%).

The use of AEDs was reported in 269/992 (27%) (Table 1), and 93/269 (43%) reported taking more than one AED. AED use was greater in adults (88%) than in children (7%); p < 0.001 and in those with focal seizures (77/167, 46%) than in those with generalized seizures (192/825, 23%; p < 0.001).

### Causes of hospital admissions among PWE

#### Epilepsy‐related causes

Overall 540/992 (54%) of the admissions were related to epilepsy. CSE was reported in 376/992 (38%) (Table 1), being more common in adults than in children (p = 0.005), but did not differ between males and females (p = 0.838). CSE was more common in those who experienced focal (80/167, 48%) than generalized seizures (296/825, 36%; p = 0.003) and occurred more in nonfebrile (260/639, 41%) compared to febrile admissions (116/353, 33%; p = 0.020). CSE occurred more in those with a history of AED use (>90% being on phenobarbital) (120/269, 45%) than in those without (256/723, 35%; p = 0.008). Postictal coma was documented in 122/992 (12%) (Table 1), and occurred more in children than adults: p = 0.010. Postictal coma was less common in those with CSE (26/376, 7%) than in those without (96/616, 16%; p < 0.001). Epilepsy‐related psychosis was documented in 10/992 (1%), being similar in children and adults (p = 0.28). Postictal psychosis occurred more in those with focal seizures (6/167, 4%) than generalized seizures (4/825, 1%; p = 0.006).

Burns were reported in 55/992 (6%) (Table 1), being more common in adults than children (p < 0.001), but were similar in females and males (p = 0.98). Burns were more common in those with a history of AED use (29/269, 11%) than in those without (26/763, 3%; p < 0.001). Accidents from falls were documented in 12/992 (1%) (Table 1) and were more common in adults than children; Fisher's exact p < 0.001. Accidents occurred more in those with a history of AED use (9/269, 3%) than those without (3/763, <1%; p < 0.001).

#### Admissions unrelated to epilepsy

Febrile illnesses were reported in 353/992 (36%), occurring more in children than adults; p < 0.001. Other less common causes of admission unrelated to epilepsy included vomiting, poisoning, hypertension, skin problems, and hydrocephalus, and their proportions are shown in Table 1.

### Duration of hospitalization

The overall median duration of hospitalization in days was 3.0 (IQR 1.0–6.0) days (Table 1) and was lower in children than in adults; p < 0.001. The overall prolonged hospital stay (>75th percentile of 6 days) was 188/992 (19%), and the proportion in adults was significantly greater than in children (67/249 [27%] vs. 121/743 [16%], p < 0.001). Prolonged hospital stay was more common in those with epilepsy‐related complications than in those without (121/540 [22%] vs. 67/452 [15%], p = 0.002).

Several epilepsy factors were associated with prolonged hospital stay in the univariate analysis (Table 3). In the multivariable analysis, prolonged duration of hospitalization independently increased with older age (OR 1.02, 95% CI 1.02–1.04; p = 0.010).

**Table 3:** Univariate analysis of factors associated with prolonged hospital stay and mortality

Feature	Prolonged hospital stay		Mortality	
	Odds ratio (95% CI)	p‐Value	Odds ratio	p‐Value
Age in years	1.02 (1.01–1.04)	<0.001	1.06 (1.04–1.08)	<0.001
Male sex	1.12 (0.81–1.55)	0.483	0.65 (0.30–1.41)	0.278
AED use	1.75 (1.25–2.45)	0.001	2.20 (1.02–4.77)	0.045
Febrile illnesses	1.29 (0.93–1.79)	0.124	1.07 (0.48–2.36)	0.873
Nonmalaria illnesses	1.75 (0.78–3.93)	0.1720	1.40 (0.18–10.60)	0.745
Respiratory tract infections	1.33 (0.80–2.20)	0.273	–	–
Focal seizures	1.44 (0.97–2.14)	0.072	1.13 (0.42–3.02)	0.813
Convulsive status epilepticus	0.91 (0.66–1.27)	0.586	0.36 (0.14–0.97)	0.043
Postictal coma	0.82 (0.49–1.36)	0.442	3.14 (1.35–7.35)	0.008
Psychosis	0.47 (0.06–3.75)	0.478	4.09 (0.50–33.44)	0.189
Accidents	1.43 (0.38–5.34)	0.593	–	–
Other causes^a^	1.89 (0.94–3.79)	0.073	4.49 (1.48–13.68)	0.008

OR, odds ratio; CI, confidence interval; AED, antiepileptic drug.

a Other causes included undetermined causes or others such as poisoning, diarrhea and vomiting, hypertension, and skin infections. Prolonged hospital stay was defined as >75th percentile of duration of stay in hospital, which was ≥6 days. There were few numbers to run the analysis for those with burns.

### Case fatality

The overall case fatality among PWE was 27/992 (3%) (Table 1), and was higher in adults than children (p < 0.001). Case fatality was similar in those with epilepsy‐related complications and those without (15/540 [3%] vs. 12/452 [3%], p = 0.91). Several epilepsy factors were associated with overall mortality in the univariate analysis (Table 3). In the multivariable analysis, mortality was independently associated with older age (OR 1.07, 95% CI 1.04–1.10; p < 0.001).

## Discussion

We have reported the first data from linked clinical and demographic surveillance systems on the burden of hospital use among PWE in sub‐Saharan Africa. The incidence of use of hospital services is high, particularly in children, but reduced in the last 3 years. There are substantial DALYs (3.1/1,000 PYO) associated with hospitalized epilepsy. Epilepsy‐related complications such as CSE and postictal coma accounted for 55% of admissions among PWE. PWE spend considerable time in hospital and mortality is common, with risk increasing with age.

### Incidence and hospital use

The incidence of hospital admission of patients with epilepsy in our study is similar to that reported in developed countries such as the United States^6^ and the United Kingdom,^4^ where admissions were compared between the early 1990s and 2000s. Epilepsy‐related complications accounted for more than half of admissions among PWE, much higher than in America (41%).^4^ The observed incidence of admission adds a significant burden to rural hospitals, which are already under‐resourced, given that epilepsy requires specialized management. The incidence remained high in the first 6 years, but appeared to decrease thereafter.^13^

The incidence of hospital use among PWE is higher than that for several other conditions in the same hospital^23^, ^24^; the difference could be ascribed to setting‐up of an epilepsy clinic within the admitting hospital and sensitization by health education programs.^13^ In addition, the high incidence may be caused by complications related to untreated epilepsy due to unavailability of AEDs in the community clinics, particularly phenobarbital.^25^ The incidence was high in children who have the least adherence to AEDs in this area,^8^, ^13^ which complicates epilepsy necessitating admission to hospital. Similarly, most children were males, which explains the high incidence in this group, although sex bias of admission cannot be ruled out.

### The burden of hospitalization

The burden of epilepsy on hospital services in this area, as measured by DALYs, is comparable to that for admissions with meningitis (3.9/1,000 PYO) and diabetes (3.6/1,000 PYO), greater than that for stroke (1.7/1,000 PYO), but lower than that for HIV (20.5/1,000 PYO).^23^ The DALYs are comparable with those of the GBD study (5.6/1,000 population) and a population‐based study in this area (4.3/1,000 population), which are based on community samples.^26^ YLD contributes more than half of the DALYs (55%) in our study but only 26% in a community study,^26^ since most hospitalized epilepsy is severe and does not include deaths that occur in the community. Disabilities associated with epilepsy are increasingly being recognized,^27^ and DALYs, which represent the true burden of epilepsy, should be the basis for economic analysis of epilepsy management.

### Factors associated with hospitalization

History of AED use was associated with hospitalization, suggesting that AEDs are used by those with severe epilepsy who would visit this district hospital for treatment or admission. This hypotheses is supported by the preponderance of focal features in those using AEDs, who also experienced significantly more frequent seizures and status epilepticus in a previous study.^8^ Previous hospitalization was associated with hospital use, probably because these people live near hospitals and are likely to seek care even for nonepilepsy causes such as acute encephalopathy. Adverse perinatal events could be associated with severe focal epilepsy that will require frequent hospitalization.

### Causes of admission

CSE was the most important cause of admission, and is a common complication of epilepsy in SSA, where it is associated with considerable mortality and sequelae.^8^, ^28^ It is likely that CSE occurred in those patients with severe epilepsy, since it was common in AED use, which was associated with focal features. Postictal coma was an important cause of admission and it was likely seizure related and could reflect high focal epilepsy (temporal and frontal lobe).^29^ Burns and accidents were increased in those with a history of AED use, suggesting noncompliance in the community. Infections were more important non–epilepsy‐related causes of admission in children than adults, as expected.

### Hospital stay and mortality

PWE were more likely to have prolonged stays in the hospital if an epilepsy‐related cause was documented, compared to those without such causes. Prolonged hospital stay is of public health importance, since it increases the costs of managing epilepsy.^30^ These costs can be twice as high in children as in adults^6^ because of significantly higher burden of admission in the former. A public health plan that takes into account estimated costs and treatment options for PWE in this area is required.^31^

The hospital case‐fatality proportions are twice those reported in a health care cost and utilization project in the United States.^6^ This discrepancy is explained by the resource‐strained hospital used in our study, with limited capacity to manage epilepsy‐related complications such as status epilepticus,^8^ a medical emergency associated with mortality.^32^ Mortality occurred more in adults than in children, probably explained by the cumulative risk of dying with age and the underlying causes of epilepsy in old age.^33^ AED use was not independently associated with mortality in this study (small sample size for deaths), but availability and adherence to AED may reduce mortality.^34^

### Strengths and limitations

This is the first study to provide the incidence of hospitalized epilepsy and the burden it impacts on a rural district hospital using data from linked clinical and demographic surveillance system. These data can be used to plan health services in this and other similar hospitals in SSA.

We were not able to estimate the burden of epilepsy in peripheral clinics or include patients who died before arrival to hospital. The risk factors for hospitalization analysis is based on randomly selected PWE for whom the background risk factor variables were available in a community survey. Neurophysiology and imaging data were not available during admission to identify focal features and grade severity. Effect of residual confounders not reported in this study such as HIV status on duration of admission and mortality cannot be excluded.

## Conclusions

The burden of hospitalized PWE is high and should be taken into account when planning for health services. Hospitalization in patients with epilepsy is attributed to severe epilepsy, probably due to delays in seeking treatment, epilepsy‐related complications such as CSE, and febrile infections. There is considerable duration of admission and high mortality, both of which could be reduced by effective and timely management of epilepsy and other nonepilepsy complications such as infections in the community. Future studies are required on the economic burden of epilepsy for planning health services. These future studies should also focus on implementation of public health interventions aimed at encouraging early initiation of treatment and adherence to prevent complications of epilepsy and subsequent hospitalization.

## Disclosure

The authors declare no conflicts of interest. We confirm that we have read the Journal's position on issues involved in ethical publication and affirm that this report is consistent with those guidelines.
